# Antimicrobial prescribing patterns at a South African tertiary referral hospital: Insights from three global point prevalence surveys

**DOI:** 10.1017/S0950268826101228

**Published:** 2026-03-02

**Authors:** Lucien Sher, Veshni Pillay-Fuentes Lorente, Jantjie Taljaard, Ann Versporten, Ines Pauwels, Erika Vlieghe, Heather Finlayson

**Affiliations:** 1 Stellenbosch University Faculty of Medicine and Health Sciences, South Africa; 2 Division of Clinical Pharmacology, Department of Medicine, Stellenbosch University Faculty of Medicine and Health Sciences, South Africa; 3 Division of Infectious Diseases, Department of Medicine, Stellenbosch University Faculty of Medicine and Health Sciences, South Africa; 4 Global Institute, Department of Family Medicine and Population health, Faculty of Medicine and Health Sciences, University of Antwerp, Belgium; 5 Laboratory of Medical Microbiology, Vaccine & Infectious Disease Institute (VAXINFECTIO), Faculty of Medicine and Health Sciences, University of Antwerp, Belgium; 6 Department of General Internal Medicine, Infectious Diseases and Tropical Medicine, University Hospital Antwerp, Belgium; 7 Department of Paediatrics and Child Health, Stellenbosch University Faculty of Medicine and Health Sciences, South Africa

**Keywords:** Antibiotic use, antimicrobial resistance, Antimicrobial stewardship, point prevalence study, AWaRe

## Abstract

Antimicrobial resistance (AMR) is a pressing global health challenge, with sub-Saharan Africa experiencing the highest burden of AMR-related deaths. Inappropriate prescribing and rising antibiotic consumption drive AMR, while limited local data hampers antimicrobial stewardship efforts. This study analysed Global Point Prevalence Survey of Antimicrobial Consumption and Resistance (Global-PPS) data from Tygerberg Hospital to identify antimicrobial use trends and inform stewardship priorities. Standard Global-PPS methodology was employed at three distinct time points. All inpatients prescribed at least one antimicrobial on the day of each survey were included in the analysis. Among 3,524 hospitalized patients, 25.9% (911/3,524) received antimicrobial therapy. Overall antimicrobial use decreased significantly (*p < 0.05*), with the largest reduction among paediatric patients (*p < 0.01*). Community-acquired infections accounted for the majority of prescriptions (50.7%; 483/952) and empirical antibiotic use was high (85.3%, 872/1022). ‘Access’ antibiotics constituted 62.7% (750/1196) of prescriptions. Single-dose prescriptions for surgical prophylaxis accounted for 17.6% (15/85). This study demonstrates progress in stewardship, particularly among paediatric inpatients. Ongoing monitoring of broad-spectrum antibiotic use and adherence to single-dose surgical prophylaxis guidelines are essential priorities. Continued Global-PPS surveillance is crucial to track trends and guide future AMS interventions.

## Introduction

Antimicrobial resistance (AMR) has emerged as one of the foremost global health threats, undermining antibiotic efficacy and decades of progress in treating infectious diseases [1, 2]. In 2019, it was estimated that 4,95 million deaths were associated with bacterial AMR, the highest burden noted in sub-Saharan Africa [3, 4]. Worldwide antibiotic consumption has increased by 46% since 2000 [5]. Escalating antimicrobial utilization and inappropriate prescribing contribute to increased resistance rates and substantial economic losses [6]. The repercussions of AMR therefore pose a formidable challenge to the sustainability of the global public health response to infectious diseases, which are the leading cause of morbidity and mortality in both adults and children [7].

Global awareness of AMR has prompted coordinated international efforts to address this rising threat. The World Health Organization (WHO) developed an AWaRe Classification of Antibiotics in 2016, a valuable instrument for overseeing antibiotic utilization, establishing objectives and assessing the impact of stewardship interventions [8, 9]. A 2023 target was set for narrow-spectrum (‘Access’) antibiotics to comprise 60% of national antibiotic prescriptions. In September 2024, the United Nations General Assembly hosted a high-level meeting where they declared ambitious targets: reducing global AMR-related deaths by 10% by 2030 and increasing the target for narrow-spectrum antibiotics to constitute 70% of national antibiotic consumption [8, 10].

Antimicrobial stewardship (AMS) optimizes therapeutic usage by mitigating the emergence of resistant pathogens without compromising access to effective treatments [11]. Robust hospital AMS programmes have demonstrated improved patient outcomes as well as reduced treatment costs and resistance rates [12–14]. Comprehensive surveillance data at both the country and hospital levels are essential for monitoring prescription trends, detecting novel resistant strains, comparing practices and evaluating the impact of interventions [2]. Initiated in 2015, the Global Point Prevalence Survey of Antimicrobial Consumption and Resistance (Global-PPS) is a standardized international surveillance initiative established to support these goals. Repeated use of point prevalence studies allows for the identification of trends in antimicrobial use and enables ongoing monitoring of the effectiveness of stewardship interventions [12].

Tygerberg Hospital (TBH) in Cape Town, South Africa has participated in the Global-PPS since 2015. Longitudinal trends in antimicrobial prescribing at TBH have not been comprehensively analysed, limiting insight into stewardship impact. This study therefore aims to describe prescribing patterns across three time points and identify key targets for AMS interventions.

## Methods

### Setting

The Global-PPS was conducted at TBH in February 2015, September 2021, and May 2022 according to standardized methodology as outlined by the Global-PPS initiative [15]. As the second-largest hospital in SA, TBH operates 1,384 active beds, including 331 paediatric beds. All patients admitted at 08 h00 on the day of the survey and receiving at least one antimicrobial agent were included in the study. All inpatient paediatric and adult wards were assessed which comprised of medical, surgical, and intensive care units (ICUs). In accordance with the WHO Anatomic Therapeutic Classification (ATC) system [16], we focused on antibiotics prescribed for systemic use. Antituberculosis drugs and antivirals were excluded from analysis, as this study focused specifically on systemic antibacterial prescribing captured on institution-specific antibiotic prescription charts. The WHO AWaRe classification system, which excludes agents, such as antifungals and antiparasitic therapies, was utilized to further categorize prescriptions [9].

### Data collection

Clinicians from various departments including paediatrics, medicine, and microbiology were trained in data collection procedures. Infection control practitioners assisted in the 2022 survey. On the day of each survey, clinicians were paired and randomly assigned wards. Uncertainty during the data capturing process was discussed with a second clinician. Standardized collection forms were used to collect data at the ward and patient level. At ward level, the total number of beds in each ward, the total number of patients admitted to each ward (denominator), and the total number of patients prescribed antibiotics (numerator) were determined. The following patient-specific data was collected: basic patient characteristics including age, gender, diagnosis, antimicrobials prescribed, therapeutic indication, and route of administration. Prescriptions were categorized as therapeutic or prophylactic. Therapeutic indications were further classified as either community-acquired infections (CAIs) or hospital-acquired infections (HAIs). HAIs were defined as new symptoms starting ≥48 h after hospital admission. Prophylactic indications were regarded for either surgical or medical purposes. Quality markers of antimicrobial prescription were also recorded, including local standard treatment guideline compliance, duration of prescription for surgical prophylaxis, presence of the reason for prescription, and documented stop or review date provided. Survey days in surgical wards were aligned with recent operative activity to ensure meaningful evaluation of surgical prophylaxis duration. Antimicrobial prescriptions were classified as biomarker-guided where documentation indicated that a laboratory biomarker (e.g. C-reactive protein or white blood cell count) informed prescribing decisions.

The collected patient data was anonymized and de-identified by assigning a unique survey number to each patient prescribed an antimicrobial. Consequently, patient consent was not required. The generated survey number was linked to the patient during data entry into the web-based Global-PPS application, developed by the University of Antwerp, Belgium. Internal validation steps identify data anomalies before an Excel spreadsheet is exported for analysis. Following validation, the administrator processed a request for real-time feedback. This study received ethical approval from Stellenbosch University Health Research Ethics Committee (Ethics Reference Number: U23/10/294).

### Data analysis

Rates were expressed as the percentage of patients on antimicrobials or as the proportional use of antimicrobial prescriptions. HAI rates were calculated as the proportion of patients with infections identified ≥48 h after admission relative to the total number of patients surveyed. For analyses of indications for antimicrobial use, duplicate antimicrobial prescriptions issued to the same patient for the same clinical indication were combined so that each patient contributed a single observation per indication. AWaRe classification analyses were restricted to antimicrobial prescriptions with an applicable AWaRe designation.

Data were grouped by indication and ward type before analysed using R and R packages (version 2024.04.1 + 748, © 2009–2024 Posit Software, PBC). In cases where continuous data follow a normal distribution, they are reported as means accompanied by standard deviations. Chi-square tests were used for comparative data analysis with significance regarded as *p* < 0.05.

## Results

### Participant characteristics

There were 3,524 hospitalized patients over the three surveys. Twenty-five per cent (911/3524) were receiving antimicrobial therapy (Table 1). The majority (71.7%; 653/911) were admitted to adult wards, whilst 21.2% (193/911) were admitted to paediatric wards and 7.1% (65/911) to neonatal wards.

**Table 1.** Overall antibiotic prevalence and distribution of treated patients across ward types for each Global-PPS survey

Characteristic	2015	2021	2022	Total	*P*-value
Total no. of hospitalized patients	1,156	1,157	1,211	3,524	0.43
Adult wards, *n* (%)	837 (72.4)	915 (79.1)	939 (77.5)	2,691 (76.3)	0.16
Paediatric and neonatal wards, *n* (%)	319 (27.6)	242 (20.9)	272 (22.5)	833 (23.6)	< 0.05
Total no. of patients receiving antibiotics, *n* (%)	332 (28.7)	294 (25.4)	285 (23.5)	911 (25.9)	< 0.05
Adult wards, *n* (%)	215 (25.7)	226 (24.7)	212 (22.6)	653 (24.3)	0.54
Medical wards, *n* (%)	70 (32.6)	53 (23.5)	46 (21.7)	169 (25.9)	< 0.01
Surgical wards, *n* (%)	112 (52.1)	153 (67.7)	132 (62.3)	397 (60.8)	< 0.01
ICU wards, *n* (%)	33 (15.3)	20 (8.8)	34 (16)	87 (13.3)	< 0.05
Paediatric and neonatal wards, *n* (%)	117 (36.7)	68 (28.1)	73 (26.8)	258 (31)	< 0.001
Medical wards, *n* (%)	89 (76.1)	53 (77.9)	53 (72.6)	195 (75.6)	< 0.05
Surgical wards, *n* (%)	6 (5.1)	10 (14.7)	4 (5.5)	20 (7.8)	0.19
Intensive care wards, *n* (%)	22 (18.8)	5 (7.4)	16 (21.9)	43 (16.6)	< 0.05

### Prevalence of antimicrobial use

There was a significant decrease in the overall prevalence of antimicrobial use between February 2015 (28.7%) and May 2022 (23.5%) (*p = 0.015*) (Table 1). Analysis by population subgroup revealed a significant reduction in the prevalence of antimicrobial use among paediatric and neonatal wards (*p* < 0.001). Across all three surveys, the highest rates of prescription were seen in the ICUs (57.4%; 132/130) followed by medical (26.6%; 408/1535) and surgical wards (25.1%; 441/1759). This was true for both adult as well as paediatric and neonatal wards.

Of patients receiving antibiotics, 24.7% (161/653) of adults and 45% of (116/258) paediatric and neonatal patients were receiving more than one antibiotic at the time of the survey. In the adult population, 19.4% (127/653) of individuals received two prescriptions and 5.2% (34/653) of paediatric patients received three or more prescriptions. In the paediatric and neonatal population, 36% (93/258) of patients received two prescriptions and 8.9% (23/258) of patients received three or more prescriptions.

### Indications of antimicrobial prescribing

Table 2 highlights the most common indications for antimicrobial prescriptions. For indication-level analyses, 952 unique patient–indication observations were analysed, with each patient contributing a single observation per infection. Patients requiring treatment for CAIs accounted for half (50.7%; 483/952) of all prescriptions across the three time periods. HAIs accounted for 28.7% (273/952), while prophylactic and other indications (skin and soft tissue infections, abdominal sepsis, and obstetric and gynaecological infections) represented 11.9% (113/952) and 8.7% (83/952) of all indications for prescription, respectively.

**Table 2.** Summary of the most common diagnoses requiring antimicrobial therapy at each time point

2015		2021		2022	
Adults	*N* = 199		*N* = 187		*N* = 180
Diagnosis	*n* (%)	Diagnosis	*n* (%)	Diagnosis	*n* (%)
Skin and soft tissue infection	53 (26.6)	Skin and soft tissue infections	43 (23.0)	Skin and soft tissue infections	41 (22.8)
Pneumonia	41 (20.6)	Pneumonia	24 (12.8)	Pneumonia	29 (16.1)
Gastrointestinal infection	15 (7.5)	Intra-abdominal infection	21 (11.2)	Intra-abdominal infection	20 (11.1)
Intra-abdominal infection	14 (7.0)	Sepsis	17 (9.1)	Sepsis	15 (8.3)
Unknown	14 (7.0)	Central nervous system infections	13 (7.0)	Bone and joint infections	11 (6.1)

Paediatrics	*N* = 117		*N* = 69		*N* = 67
Diagnosis	*n* (%)	Diagnosis	*n* (%)	Diagnosis	*n* (%)
Pneumonia	48 (41.0)	Pneumonia	23 (33.3)	Pneumonia	25 (37.3)
Sepsis	33 (28.2)	Bacteraemia with no clear anatomical site	12 (17.4)	Bacteraemia with no clear anatomical site	8 (11.9)
Central nervous system infections	5 (4.3)	Central nervous system infections	9 (13.0)	Central nervous system infections	6 (9.0)
Bone and joint infections	5 (4.3)	Intra-abdominal infections	4 (5.8)	Gastrointestinal infections	4 (6.0)
Gastrointestinal infections	5 (4.3)	Upper respiratory tract infection	3 (4.3)	Bone and joint infections	3 (4.5)

Prescriptions for CAI and HAIs were higher in paediatric and neonatal wards than in adult wards. More than half (57.2%; 158/276) of patients in paediatric and neonatal wards were prescribed treatment for CAIs compared to 48.1% (325/676) in adult wards. For HAIs, 33.7% (93/276) of patients in paediatric and neonatal wards and 26.6% (180/676) of patients in adult wards received antimicrobials. The number of patients receiving medical prophylaxis (4.1% vs. 2.2%) and surgical prophylaxis (11.5% vs. 0.4%) were comparatively higher in adult wards compared to paediatric and neonatal wards, respectively. Although HAI rates were stable across the three time periods, higher HAI rates were noted in the paediatric population (11.2%; (93/833) compared to the adult population (6.7%; 180/2691).

### AWaRe prescribing

**Figure 1.**
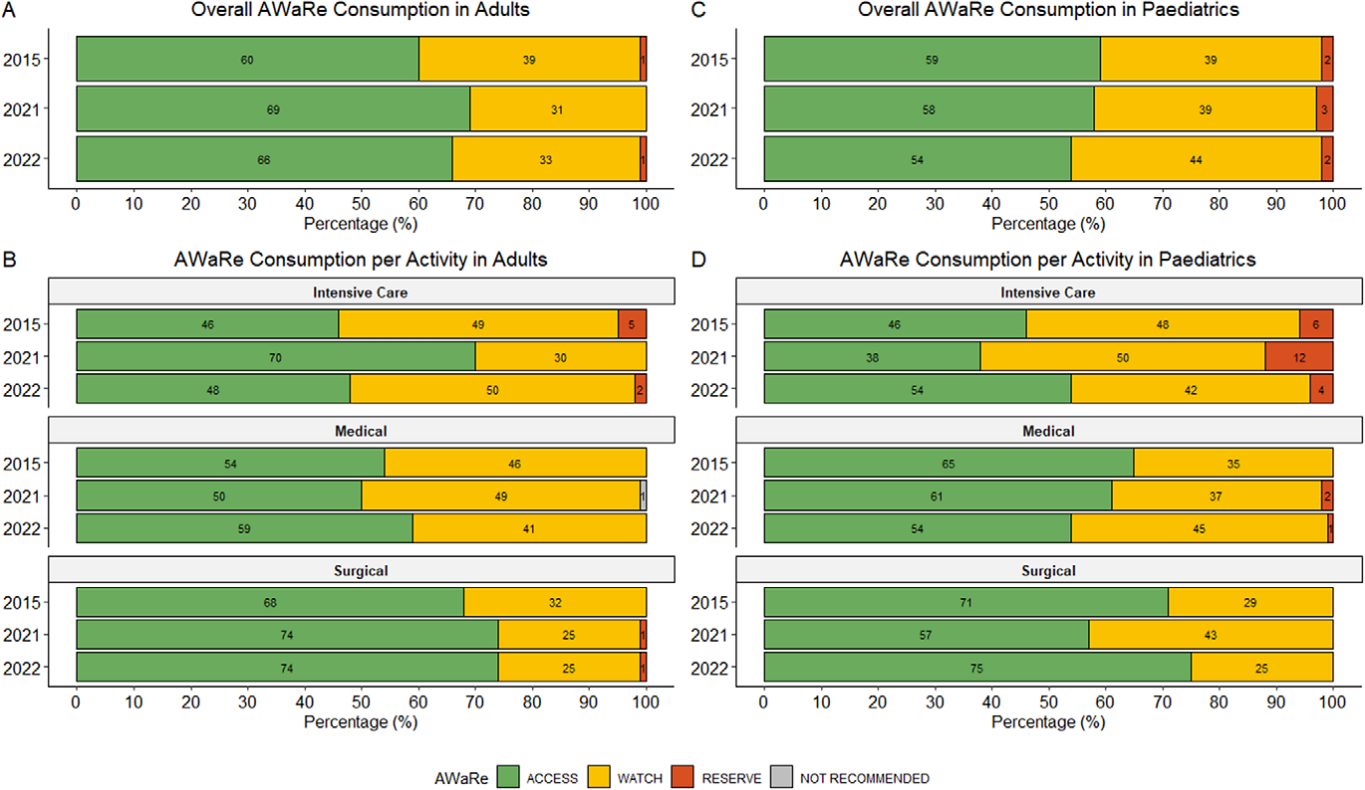
Overview of AWaRe use in the adult and paediatric populations (paediatric and neonatal wards) overall and per activity for each survey. (a) – Overall AWaRe consumption in adults at each time point. (b) – AWaRe consumption per activity in adults. (c) – Overall AWaRe consumption in paediatrics (paediatric and neonatal wards) at each time point. (d) – AWaRe consumption per activity in paediatrics (paediatric and neonatal wards). Percentages are calculated using year-specific denominators representing antimicrobial prescriptions with an applicable AWaRe classification; denominators vary by ward type and survey year.

AWaRe analyses included 1,196 antimicrobial prescriptions with an applicable AWaRe classification. Over the survey periods, 62.7% (750/1196) of prescriptions were from the ‘Access’ category, with 36.1% (432/1196) from the ‘Watch’ category and 1.1% (13/1196) from the ‘Reserve’ category. Only one prescription fell into the ‘Not Recommended’ category. In paediatric wards, the proportion of ‘Watch’ antibiotic prescriptions varied across survey years. These differences were not statistically significant overall (p = 0.688) or in paediatric medical wards (p = 0.382) (Figure 1c,d). A similar non-significant variation was observed in adult surgical wards (Figure 1b). The overall ‘Access-to-Watch’ ratio was 1.7 (750/432), with a higher index noted in the adult (1.90; 525/277) compared to paediatric population (1.45; 225/155).

#### Adults

**Figure 2.**
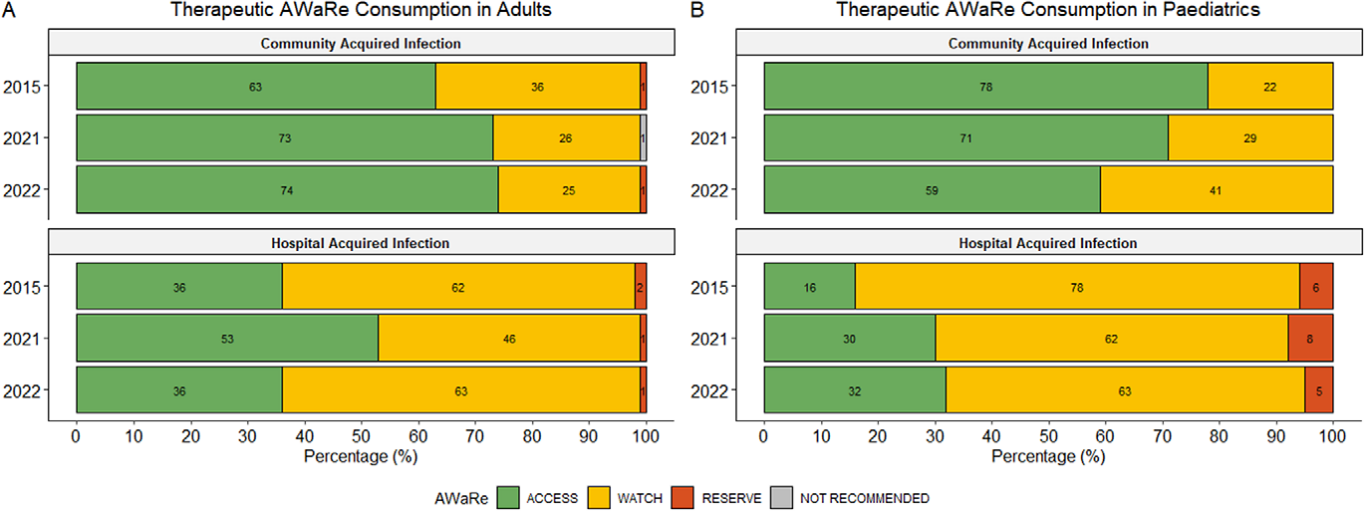
Summary of therapeutic AWaRe consumption in adults (a) and paediatrics (paediatric and neonatal wards) (b). Percentages are calculated using year-specific prescription-level denominators.

Figure 1a demonstrates the breakdown of AWaRe prescribing in the adult population. A higher percentage of ‘Access’ antibiotics were prescribed in surgical wards compared to ICUs and medical wards (Figure 1b). ‘Access’ antibiotics were more commonly used to treat CAIs, while broader-spectrum ‘Watch’ antibiotics were more frequently prescribed for the therapeutic treatment of HAIs (Figure 2a). The most commonly prescribed ‘Access’ antibiotics were amoxicillin/clavulanic acid (26.6%; 167/629), metronidazole (5.7%; 36/629), and amikacin (5.1%; 32/629). ‘Watch’ prescriptions were largely composed of ceftriaxone (10.8%; 68/629), ertapenem (4.6%; 29/629), and piperacillin with enzyme inhibitor (4.5%; 28/629). Colistin (0.5%; 3/629) and linezolid (0.3%; 2/629) were the most frequently prescribed ‘Reserve’ antibiotics in adults with both predominantly prescribed in ICUs.

#### Paediatrics

Figure 1c highlights overall AWaRe usage in the paediatric population. An increasing trend in the use of ‘Watch’ antibiotics for the treatment of CAIs was observed, though not statistically significant (*p = 0.057*) (Figure 2b). In paediatrics, a greater proportion of ‘Watch’ and ‘Reserve’ antibiotics were used to treat HAIs compared to adults. Regarding therapeutic antimicrobial use, the most prescribed ‘Access’ antibiotics in paediatrics were ampicillin (11.9%; 42/353), amoxicillin (8.2%; 29/353), and gentamicin (6.8%; 24/353). The most frequently prescribed ‘Watch’ antibiotics included meropenem (13%; 46/353), ceftriaxone (8.2%; 29/353), and vancomycin (5.4%; 19/353), while colistin (1.7%; 6/353) was the most used ‘Reserve’ antibiotic.

### Patterns of antimicrobial prescribing

The majority of all prescriptions (93.1%; 1169/1256) were for systemic antibiotics (ATC J01) (Supplementary Table 1). Supplementary Figures 1 and 2 demonstrate the most commonly prescribed ATC J01 antimicrobials prescribed for the treatment of CAIs and HAIs in adults and paediatrics, respectively. Prescriptions for systemic antimycotics (ATC J02) comprised 4.6% (58/1256) while antiprotozoals (ATC P01) and intestinal anti-infectives (ATC A07) accounted for 2.2% (27/1256) and 0.2% (2/1256) of all prescriptions, respectively (Supplementary Table 1).

**Figure 3.**
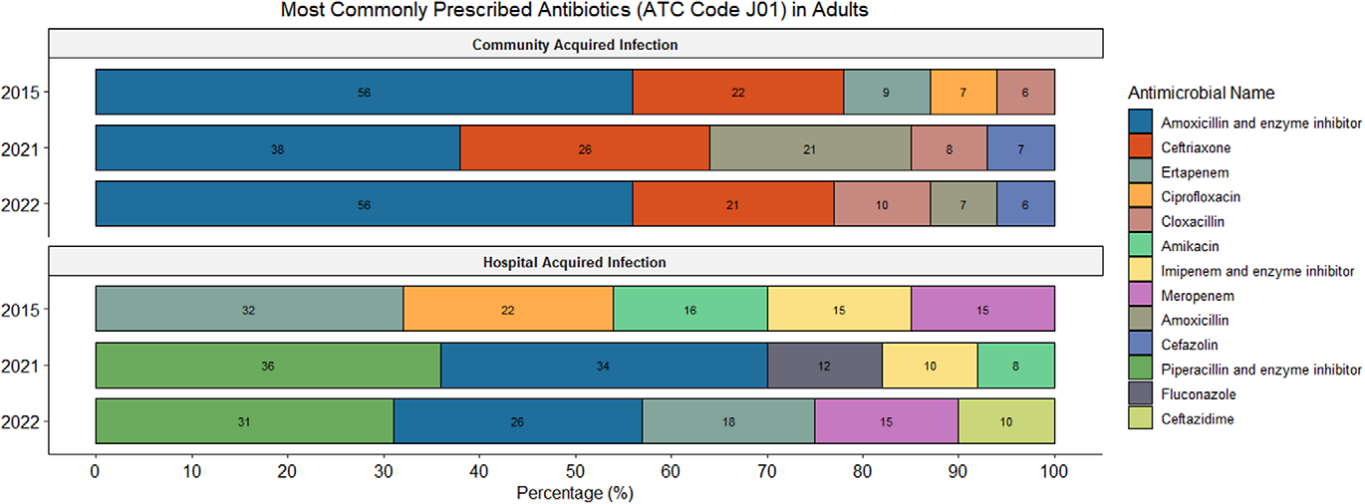
Overview of the most commonly prescribed antibiotics for therapeutic treatment (CAIs and HAIs) in the adult population.

**Figure 4.**
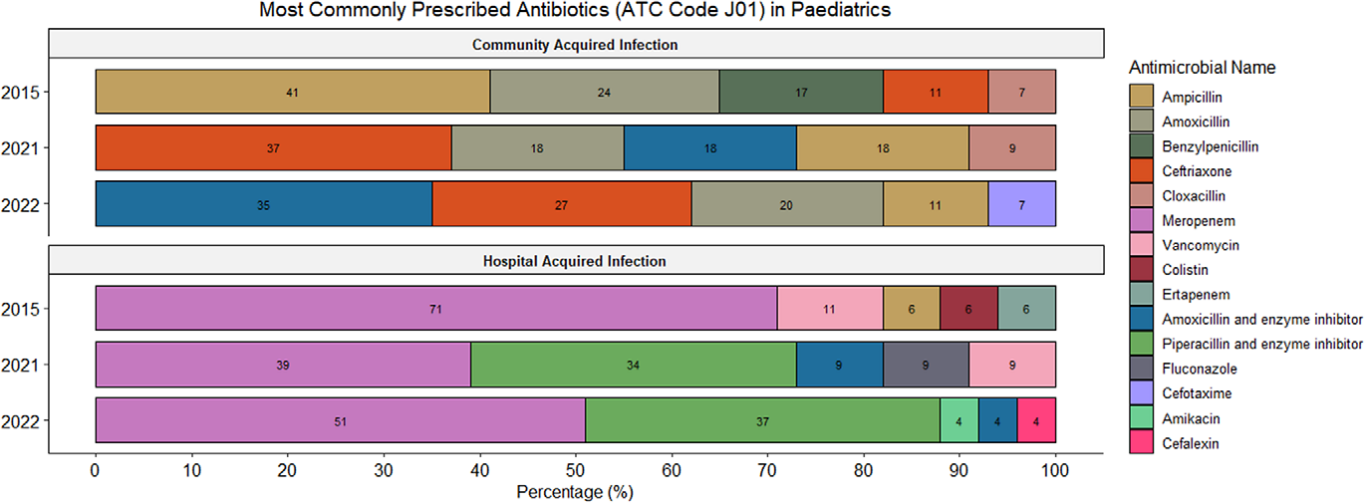
Overview of the most commonly prescribed antibiotics for therapeutic treatment (CAIs and HAIs) in the paediatric population (paediatric and neonatal wards).

Among adults overall, the most prescribed antibiotics include amoxicillin and beta-lactamase inhibitor (22.5%; 191/850), ceftriaxone (8.3%; 71/850), and cefazolin (7.7%; 65/850). In the paediatric population overall, the most prescribed antibiotics included ampicillin (13.3%; 54/406), meropenem (11.6%; 47/406), and gentamicin (8.9%; 36/406). Figures 3 and 4 summarize the most commonly utilized antimicrobials for the treatment of CAIs and HAIs in adults and paediatrics, respectively.

### Quality indicators of prescription

Quality indicator analyses were performed at the prescription level. Overall guideline compliance was high (73.4%; 922/1256). Reasons for prescribing were documented in 85.7% (1,077/1256) of cases, but only 28.4% (357/1256) included a documented stop or review date. The majority of prescriptions were empiric, including 87.3% of all prescriptions (1,097/1256), 90.2% of those for therapeutic treatment of CAI (580/643) and 77% for HAI (292/379). Among these, 33.3% of all prescriptions (418/1256), 26.9% of empiric CAI prescriptions (156/580), and 55.1% of empiric HAI prescriptions (161/292) were guided by a biomarker.

#### Adults

**Figure 5.**
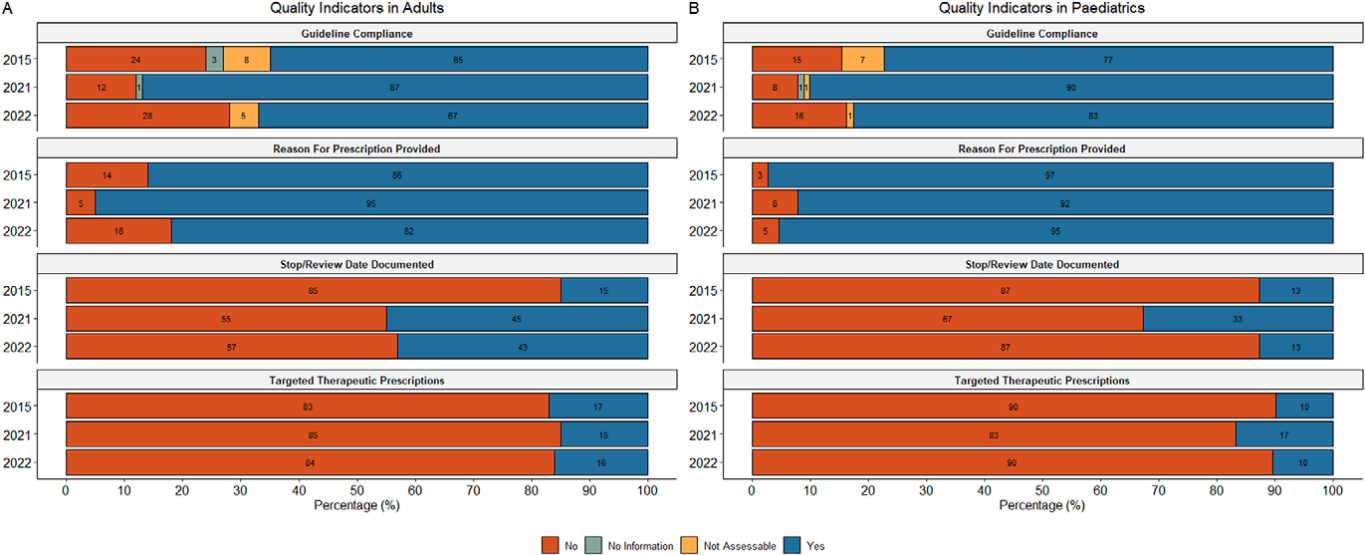
Overview of quality indicators of antimicrobial prescription in the adult (a) and paediatric (b) populations (paediatric and neonatal wards).

The adult population showed significant improvements over time in both compliance with prescribing guidelines and the proportion with a documented reason (*p < 0.001*) (Figure 5a). The documentation of stop or review dates increased from 15.2% (35/230) in 2015 to 43.2% (79/183) in 2022 (*p < 0.001*). The percentage of therapeutic prescriptions that were targeted remained low and relatively stable (*p = 0.77*) (Figure 5a). In the adult population, 72% (612/850) of therapeutic prescriptions were administered parenterally.

#### Paediatrics

Paediatric therapeutic prescriptions demonstrated a non-significant improvement in compliance with local therapeutic prescribing guidelines (*p = 0.12*) (Figure 5b). The percentage of prescriptions with a documented reason remained high, with no statistically significant changes over time (*p = 0.14*). Documentation of stop or review dates showed significant fluctuations (*p < 0.001*) with 81% (329/406) of therapeutic paediatric prescriptions given parenterally.

### Surgical antibiotic prophylaxis

In 2015, there were no single-dose surgical prophylactic prescriptions (0/16). The number of single-dose prescriptions declined from 2021 (25.6%; 10/39) to 2022 (16.7%; 5/30). The most common surgeries for which surgical prophylaxis was prescribed were skin, soft tissue, bone or joint surgery (41.2%; 35/85), gastrointestinal surgery (14.1%; 12/85), and cardiac or vascular surgery (12.9%; 11/85). Cefazolin accounted for 50.6% of all surgical prophylactic prescriptions, followed by amoxicillin/clavulanic acid (12.9%; 11/85) and ciprofloxacin (12.9%; 11/85).

## Discussion

This study evaluated antimicrobial prescribing patterns at TBH over three time points and identified an overall reduction in antimicrobial use, largely driven by decreased paediatric prescribing. Although over 60% of prescriptions fell into the WHO ‘Access’ category, a notable proportion of ‘Watch’ and ‘Reserve’ antibiotics were prescribed in ICUs and for paediatric HAIs. While guideline compliance was relatively high, documentation of stop or review dates remained suboptimal and surgical prophylaxis practices exhibited poor adherence to recommended single-dose regimens.

The paediatric prevalence of antimicrobial use rate at our institution (31.0%) compares favourably with global paediatric rates of 36.7% [17, 18] and rates observed in other low- and middle-income countries (Nigeria 68.8% and Myanmar 60.4%) [19, 20]. However, Chetty et al. reports a lower point prevalence (22.9%) among paediatric inpatients from three academic hospitals in SA [21]. This was attributed to daily consultant-led prescription review, facilitating timely de-escalation of empiric therapy [21]. The significant reduction in paediatric prescribing in our setting may reflect robust stewardship practices, including the introduction of an independent antibiotic prescription chart in 2016 and targeted weekly stewardship rounds. Additionally, the potential influence of the coronavirus disease (COVID-19) pandemic isolation measures cannot be overlooked. COVID-19 had a relatively low direct impact on paediatric populations but was associated with a marked reduction in other respiratory viral infections, such as respiratory syncytial virus and adenovirus, which are frequently treated empirically with antibiotics during the early phase of infection [22].

Skin and soft tissue infections and pneumonia remained the primary indications for antibiotic therapy in adult wards. This may be attributed to socioeconomic factors, a high prevalence of comorbidities such as diabetes mellitus and human immunodeficiency virus infection as well as a substantial burden of trauma and interpersonal violence in the SA population [23]. The prevalence of prescriptions treating CAIs (50.7%) was lower than observed in the African region (57.4%) but higher than the global rate of 45.6% [15]. This could reflect the patient demographic at TBH, a referral hospital that typically receives patients >48 h after their initial admission elsewhere.

The AWaRe classification framework allowed us to gauge progress towards WHO targets [8]. Overall, more than 60% of prescriptions fell into the ‘Access’ category, which is encouraging given the worldwide documented rise in ‘Watch’ antibiotic use globally [24]. Although AWaRe prescribing in the paediatric population aligns with point prevalence surveys targeting children and neonates globally [17, 18, 25] and in other African countries [19, 26, 27], a recent review of paediatric antimicrobial prescribing across state hospitals in SA reported an improved ‘Access-to-Watch’ ratio (2.0) compared to our study (1.45) [28].

The progressive reliance on ‘Watch’ antibiotics for the treatment of paediatric and neonatal CAIs may be partially explained by the challenge of excluding central nervous system infections in sick infants <3 months of age, who are empirically treated with ceftriaxone as first-line until meningitis is excluded. Third-generation cephalosporins remain important first-line treatment options in a number of clinical presentations, despite their association with extended-spectrum beta-lactamase-producing microorganisms, already prevalent among neonatal wards of TBH [29, 30].

A greater proportion of ’Watch’ and ’Reserve’ antibiotics were used to treat paediatric HAIs compared to adults. This may reflect the underutilization of appropriate broad spectrum ‘Watch’ and ‘Reserve’ antibiotics or higher rates of therapy de-escalation in adults, though further research is required to verify this. The increase in ‘Access’ antibiotic use in the adult group during the second survey is surprising but may reflect antibiotic protocols implemented during the COVID-19 pandemic. These data underscore the need to interpret prescribing behaviour in the context of local resistance profiles and the importance of close monitoring in subsequent surveys.

The commonly treated infections in our paediatric population align with findings of other paediatric studies [17, 18, 26–28, 31]. The increasing reliance on beta-lactam/beta-lactamase inhibitor combinations and third-generation cephalosporins could reflect updates to national paediatric pneumonia guidelines, which recommend amoxicillin/clavulanic acid as first-line treatment for severe pneumonia instead of traditional ampicillin-gentamicin combination [32]. One-third of all paediatric prescriptions were for HAIs, indicating both the vulnerability of this patient group to HAIs and the significant role these infections play in driving antibiotic use [21, 30]. Informed by hospital antibiograms, guidelines were introduced in 2016 recommending piperacillin–tazobactam in combination with amikacin as first-line therapy for HAIs in both adult and paediatric patients [33]. Additionally, an authorization policy was implemented requiring approval from specialist consultants or infectious disease specialists for the prescription of ‘Watch’ and ‘Reserve’ antibiotics [34].

Quality indicators of antibiotic prescribing are important for benchmarking quality improvement initiatives. While our findings align with those of a recent point prevalence survey conducted in the Limpopo province of SA [35], other African countries have reported higher rates of guideline compliance (Ghana 84% and Tanzania 88%), as well as superior documentation of stop or review dates in prescriptions (Ghana 67%, Uganda 99%, and Tanzania 99%) [36]. Compared to adults, paediatric prescriptions were more frequently guideline compliant, although both groups demonstrated poor documentation of stop or review dates. Implementing automatic stop or review dates has been shown to improve compliance [13]. This ensures regular re-assessment of antibiotic therapy, minimizing unnecessary prolonged treatment, reducing reliance on manual processes, and lowering the risk of human error. Hospital AMS programmes require ongoing reinforcement and monitoring to enhance these quality indicators, especially in settings of high turnovers of healthcare professionals [37].

The proportion of therapeutically targeted prescriptions remained low and relatively stable across both adult and paediatric populations. This may be due to the predominant empiric treatment of CAIs in our setting. However, many empiric prescriptions were still guided by biomarker data, with over one quarter of CAIs and more than half of HAIs informed in this way. As point prevalence surveys capture data at a single timepoint, they may underestimate the number of empiric prescriptions that are subsequently refined based on diagnostic results. This underscores the critical role of diagnostics and laboratory capacity to reduce turnaround time and enable the provision of prompt targeted therapy.

Despite guidelines recommending a single, narrow-spectrum antibiotic dose within 24 h preoperatively [38], only a small proportion of prescriptions in our study followed this practice (17.6%). Prolonged prophylaxis is associated with higher resistance rates, minimal clinical benefit and adverse events [38, 39]. In 2016, Brink et al. demonstrated improvements in antibiotic choice and duration of prophylaxis through education initiatives, developing a surgical antibiotic prophylaxis toolkit and obtaining buy-in from relevant hospital stakeholders [40]. At TBH, a surgical site bundle is underway to address low single-dose adherence and ongoing monitoring will ensure alignment with best practices.

This study has several limitations inherent to its cross-sectional design. Data were partially collected during the COVID-19 pandemic in 2021 and 2022, limiting the ability to assess temporal trends in antimicrobial use. This timing may have influenced prescribing patterns through changes in the types and severity of patients admitted, service delivery and infection epidemiology. Consequently, comparisons across survey years should be interpreted cautiously. The absence of surveys between 2015 and 2021 reflects logistical and resource constraints rather than a deliberate sampling strategy. Additional limitations include reliance on medical record documentation and the inability to assess prescribing appropriateness or treatment duration. Combining paediatric and neonatal populations may also have obscured age-specific patterns.

## Conclusion

This study showed a reduction in prevalence of antimicrobial use over three time periods, particularly among paediatric and neonatal patients, reflecting progress in AMS efforts. However, high HAI rates are concerning and highlight the importance of infection prevention control in minimizing HAIs and resultant antibiotic use. Enhancing guideline compliance, optimizing surgical prophylaxis, and reinforcing targeted prescribing practices will be critical to mitigating resistance risks. Future interventions should prioritise adult wards, focusing on senior prescription reviews, optimized surgical prophylaxis and close monitoring of ‘Watch’ and ‘Reserve’ antibiotic use. These efforts should be complemented by post-intervention surveys to monitor progress and ensure alignment with national and global stewardship targets.

## Supporting information

10.1017/S0950268826101228.sm001 Sher et al. supplementary material Sher et al. supplementary material

## Data Availability

The data supporting this study are part of the Global Point Prevalence Survey (Global-PPS) and are not publicly available due to institutional and data governance policies. Anonymized datasets are securely stored at the University of Antwerp. Access may be granted upon reasonable request and with approval from both the Global-PPS coordinating team and Stellenbosch University. Please contact the corresponding author (finlayson@sun.ac.za) for further information.

## Abbreviations

AMR: Antimicrobial Resistance
AMS: Antimicrobial Stewardship
ATC: Anatomic Therapeutic Classification
AWaRe: ‘Access’, ‘Watch’ and ‘Reserve’ (WHO Classification of Antibiotics)
CAI: Community-acquired Infection
COVID-19: Coronavirus Disease
HAI: Hospital-acquired Infection
ICU: Intensive Care Unit
LMIC: Low- and Middle-Income Countries
SA: South Africa
TBH: Tygerberg Hospital
WHO: World Health Organization

## Supplementary material

The supplementary material for this article can be found at http://doi.org/10.1017/S0950268826101228.
