# Supplementary material for: Antimicrobial prescribing patterns at a South African tertiary referral hospital: Insights from three global point prevalence surveys
Source: Epidemiol Infect. 2026 Mar 2;154:e39. doi: 10.1017/S0950268826101228 (PMC13100929; doi:10.1017/S0950268826101228)

Supplementary Table 1. Summary of antimicrobial prescriptions according to the ATC system across multiple time points for both adult and paediatric patients (paediatric and neonatal wards).

|  |  | 2015 | | 2021 | | 2022 | |  |
| --- | --- | --- | --- | --- | --- | --- | --- | --- |
| Antimicrobial classification | **ATC Code** | **Adult** | **Paediatric** | **Adult** | **Paediatric** | **Adult** | **Paediatric** | **Total** |
| Antidiarrheals, intestinal anti-inflammatory/anti-infective agents, n (%) | **A07** | **1 (50)** | **1 (50)** | **0** | **0** | **0** | **0** | **2** |
| Antibiotics | A07AA | 1 (0) | 1 (1) | 0 (0) | 0 (0) | 0 (0) | 0 (0) | 2 |
| Antibacterials for systemic use, n (%) | **J01** | **253 (22)** | **177 (15)** | **276 (24)** | **108 (9)** | **254 (22)** | **101 (8)** | **1169** |
| Tetracyclines | J01AA | 4 (1) | 0 (0) | 2 (1) | 1 (1) | 2 (1) | 0 (0) | 9 (1) |
| Penicillins with extended spectrum | J01CA | 20 (6) | 46 (20) | 35 (9) | 22 (13) | 47 (17) | 17 (15) | 187 (16) |
| Beta-lactamase sensitive penicillins | J01CE | 0 (0) | 12 (5) | 4 (1) | 1 (1) | 1 (0) | 3 (3) | 21 (2) |
| Beta-lactamase resistant penicillins | J01CF | 12 (4) | 7 (3) | 17 (4) | 5 (3) | 11 (4) | 0 (0) | 52 (4) |
| Combinations of penicillins, including beta-lactamase inhibitors | J01CR | 79 (24) | 4 (2) | 73 (18) | 18 (11) | 68 (25) | 21 (18) | 263 (22) |
| First-generation cephalosporins | J01DB | 7 (2) | 1 (1) | 33 (8) | 0 (0) | 25 (9) | 2 (2) | 68 (6) |
| Second-generation cephalosporins | J01DC | 1 (0) | 1 (1) | 2 (1) | 0 (0) | 0 (0) | 0 (0) | 4 (0) |
| Third-generation cephalosporins | J01DD | 24 (7) | 21 (9) | 27 (7) | 15 (9) | 25 (9) | 11 (10) | 123 (11) |
| Fourth-generation cephalosporins | J01DE | 1 (0) | 0 (0) | 0 (0) | 0 (0) | 1 (0) | 0 (0) | 2 (0) |
| Carbapenems | J01DH | 31 (9) | 26 (11) | 10 (3) | 12 (7) | 18 (7) | 18 (16) | 115 (10) |
| Intermediate-acting sulfonamides | J01EC | 0 (0) | 0 (0) | 0 (0) | 0 (0) | 1 (0) | 0 (0) | 1 (0) |
| Trimethoprim and derivates | J01EA | 0 (0) | 0 (0) | 0 (0) | 0 (0) | 1 (0) | 0 (0) | 1 (0) |
| Combinations of sulfonamides and trimethoprim, including derivatives | J01EE | 9 (3) | 14 (6) | 6 (1) | 3 (2) | 5 (2) | 3 (3) | 40 (3) |
| Macrolides | J01FA | 16 (5) | 7 (3) | 8 (2) | 1 (1) | 8 (3) | 1 (1) | 41 (4) |
| Lincosamides | J01FF | 2 (1) | 0 (0) | 2 (1) | 1 (1) | 3 (1) | 0 (0) | 8 (1) |
| Aminoglycosides | J01GB | 11 (4) | 20 (9) | 24 (6) | 19 (11) | 18 (7) | 19 (17) | 111 (9) |
| Fluoroquinolones | J01MA | 22 (8) | 3 (1) | 11 (4) | 1 (1) | 4 (1) | 1 (1) | 42 (4) |
| Combinations of antibacterials | J01RA | 0 (0) | 0 (0) | 1 (0) | 0 (0) | 0 (0) | 0 (0) | 1 (0) |
| Glycopeptide antibacterials | J01XA | 5 (2) | 11 (5) | 9 (2) | 5 (3) | 9 (3) | 3 (2) | 42 (4) |
| Polymyxins | J01XB | 1 (0) | 3 (1) | 1 (0) | 2 (1) | 1 (0) | 2 (2) | 10 (1) |
| Imidazole derivatives | J01XD | 7 (2) | 1 (0) | 11 (3) | 2 (1) | 3 (1) | 0 (0) | 24 (2) |
| Nitrofuran derivatives | J01XE | 0 (0) | 0 (0) | 0 (0) | 0 (0) | 2 (1) | 0 (0) | 2 (0) |
| Other antibacterials | J01XX | 1 (0) | 0 (0) | 0 (0) | 0 (0) | 1 (0) | 0 (0) | 2 (0) |
| Antimycotics for systemic use, n (%) | **J02** | **8 (14)** | **6 (10)** | **20 (34)** | **4 (7)** | **13 (22)** | **7 (13)** | **58** |
| Antimycotics | J02AA | 1 (0) | 0 (0) | 2 (1) | 0 (0) | 2 (1) | 1 (1) | 6 (10) |
| Triazole and tetrazole derivatives | J02AC | 7 (2) | 6 (3) | 16 (4) | 3 (2) | 9 (3) | 4 (3) | 45 (78) |
| Other antifungals for systemic use | J02AX | 0 (0) | 0 (0) | 2 (0) | 1 (0) | 2 (1) | 2 (2) | 7 (12) |
| Antiprotozoals, n (%) | **P01** | **13 (48)** | **2 (7)** | **7 (26)** | **0 (0)** | **5 (19)** | **0 (0)** | **27** |
| Nitroimidazole derivatives | P01AB | 12 (4) | 2 (1) | 7 (2) | 0 (0) | 5 (2) | 0 (0) | 26 (96) |
| Artemisinin and derivatives | P01BE | 1 (0) | 0 (0) | 0 (0) | 0 (0) | 0 (0) | 0 (0) | 1 (4) |

Supplementary Figure 1. Summary of the most common ATC J01 antimicrobials used to treat CAIs and HAIs in the adult population. ATC – Anatomic Therapeutic Classification system.


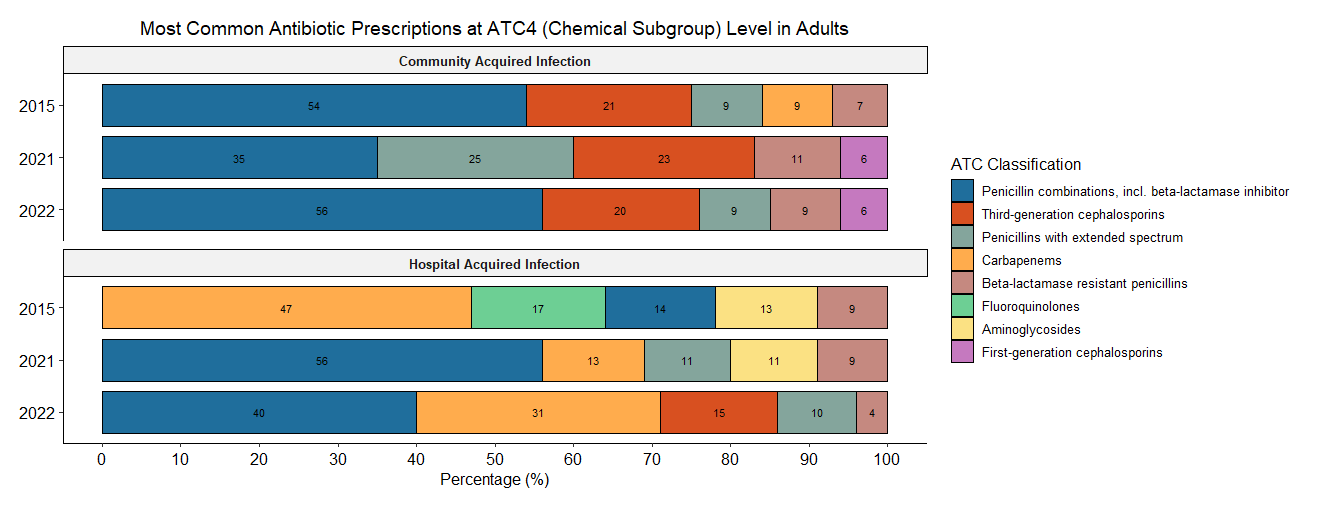


Supplementary Figure 2. Summary of the most common ATC J01 antimicrobials used to treat CAIs and HAIs in the paediatric population (paediatric and neonatal wards). ATC – Anatomic Therapeutic Classification system


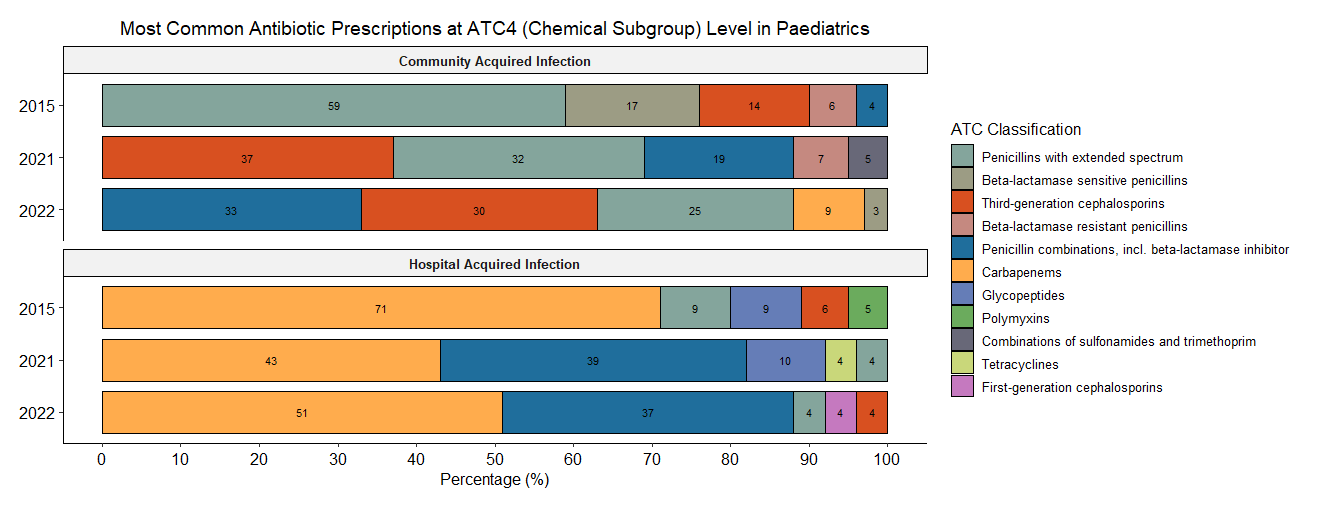

Supplement: Sher et al. supplementary material [file S0950268826101228sup001.docx]
